# Involvement of TNFα, IL-1β, COX-2 and NO in the anti-inflammatory activity of *Tamarix aphylla* in Wistar albino rats: an in-vivo and in-vitro study

**DOI:** 10.1186/s12906-024-04359-8

**Published:** 2024-01-25

**Authors:** Nada Fayez, Waleed Khalil, Essam Abdel-Sattar, Abdel-Fattah Mohamed Abdel-Fattah

**Affiliations:** 1 Pharmacology Department, Faculty of Veterinary Medicine, Suez Canal University, Ismailia, 41522 Egypt; 2 Pharmacognosy Department, Faculty of Pharmacy, Cairo University, Kasr El- Aini St, Cairo, 11562 Egypt

**Keywords:** *Tamarix aphylla*, Anti-inflammatory, Carrageenan, Diclofenac, Paw edema

## Abstract

**Background:**

With the emergence of many side effects from synthetic drugs, there is an urgent need to find a natural alternative to these products. Therefore, our primary aim was to evaluate the anti-inflammatory activity of *Tamarix aphylla* (TA) and investigate the potential mechanism underlying this action.

**Methods:**

Initially, to ensure the safety of the extract and for dose selection, we performed an acute oral toxicity Assay through the oral administration of graded doses up to 4 g\kg in Wistar rats. then, we used the carrageenan-induced edema model to elucidate the anti-inflammatory activity. Using specific ELISA kits, we measured the levels of TNF-α, IL-1β, COX-2 and NO inside the inflamed paw tissue. Finally, for the *in-vitro* anti-inflammatory experiment, we used the erythrocyte membrane stability test.

**Results:**

Based on the acute oral toxicity assay, *T. aphylla* was considered generally safe and three different doses of 100, 200, and 400 mg/kg were chosen for further experiments. Additionally, TA expressed a significant (*P* < 0.05) anti-inflammatory activity, showing the maximum inhibition percentage at the fifth hour of measurement at 53.47% and 70.06%, at doses of 200 and 400 mg/kg respectively, compared to 63.81% for the standard drug. Similarly, we found that TA effectively reduced the levels of TNF-α and IL-1β at all tested doses (100-200-400 mg/kg) to a greater extent than the standard drug. Moreover, at 400 mg/kg, TA was able to significantly lower the levels of COX-2 and NO inside the inflamed tissue to a level comparable (*P* < 0.05) with that measured inside the paw tissue of normal rats. Finally, *Tamarix aphylla* at 100, 200 and 400 mg/kg doses significantly (*P* < 0.05) inhibited the heat-induced hemolysis of RBCs membrane by 67.78, 74.82 and 82.08%, respectively, compared to 83.89% produced by Aspirin.

**Conclusion:**

T. aphylla produced a significant (*P* < 0.05) anti-inflammatory activity compared to the standard drugs either through the reduction of pro-inflammatory mediators or the protection of the lysosomal membrane.

**Supplementary Information:**

The online version contains supplementary material available at 10.1186/s12906-024-04359-8.

## Background

Inflammation is a non-specific process that is regulated by the body as a protective mechanism against tissue injury or infection. While inflammation is considered a normal physiological event, it can lead to serious pathological complications or worsen major diseases if homeostatic control is disrupted [1, 2]. Several markers have a crucial role in inflammation progression including cytokine receptors, nitric oxide (NO), tumor necrosis factor-alpha (TNF-α), chemokines, interferons and pro-inflammatory enzymes such as cyclooxygenase (COX-2) [3, 4]. All these mediators are considered the main targets of the anti-inflammatory agents. Among these cytokines, TNF-α is a common pro-inflammatory cytokine released during acute inflammatory processes. Other contributors to inflammation are IL-1β, COX-2 and NO [5–7].

Regrettably, there are many emerging side effects due to the frequent use of synthetic drugs. Side effects range from minor symptoms to life-threatening ones. Annually, thousands of patients die from apparently safe over-the-counter medication [8, 9]. On top of that, around 8% of American hospital admissions are due to manufactured drugs ‘side effects. That’s why, there is a renewed interest in complementary medicine and people each year back to it, believing that herbals are free from any side effects [10].

*Tamarix aphylla* (TA), Athel pine, is an evergreen tree which belongs to Tamaricaceae family. It is natively found in Asia, North Africa and southeastern Europe [11]. In folk medicine, T. aphylla has many values, anti-oxidant, anti-microbial diuretic, anthelmintic, internal tumors, inflammation and joint pains [12]. Furthermore, Many previous investigations were carried out on TA plant indicating the presence of many pharmacological and therapeutic activities such as antipyretic, analgesic, antirheumatic, anti-inflammatory [13, 14] hepatoprotective, antioxidant [15, 16], and antimicrobial [17, 18].

Nevertheless, most of the information regarding the safety and effectiveness of TA as an anti-inflammatory plant is still based on its historical beliefs. Besides, the current experimental studies on the leaves, to detect their pharmacological and therapeutic values, are not enough. Therefore, our study aimed to detect to what extent the extract is relatively safe and to assess its anti-inflammatory activity.

## Materials

### Plant collection and identification

Aerial parts of *Tamarix aphylla L.* were collected from medium-aged trees from El-Nubaria city, 75 km Cairo-Alexandria desert road, Egypt, in March 2020. The plant was identified by Agriculture Engineer Mrs. Therese Labib, Senior Botanist, Orman Botanic Garden, Giza, Egypt. The plant name was verified with the plant list (http://www.theplantlist.org/). Voucher specimens of *T. aphylla* L. (Sp. # TA 3.4.2020) were deposited at the Department of Pharmacognosy, Faculty of Pharmacy, Cairo University, Egypt. The plant was thoroughly cleaned using water and dried under shade.

### Chemicals and drugs

Carrageenan λ (Sigma Lambda, USA) and diclofenac (Declophen®) were purchased from Pharco Pharmaceutical (Alexandria, Egypt), ELISA kits (TNFα and IL-1β) were purchased from the ABclonal company (Immuno-Biological Laboratories (IBL)-America, Minneapolis, USA, catalogue number: RK00029). Rat Cyclooxygenase 2 (COX2) ELISA Kit (catalogue number: MBS020734) and Nitric Oxide Microplate Assay Kit, (catalogue number: MBS8243214) were purchased from MYBIOSOURCE company.

### Animals

Wistar albino rats (140–200 g) were obtained from the animal house at the Faculty of Veterinary Medicine-Suez Canal University. For adaptation, the animals were given seven days. The rats were housed under standard laboratory conditions with a controlled environment of temperature (23 ± 3 °C), humidity (60% ± 10%), and a 12 h light/dark cycle, and kept in polypropylene cages in groups of five and provided with free access to standard rodent pellet diet and tap water. All experiments were done at the Pharmacology Department, Faculty of Veterinary Medicine-Suez Canal University. Animal handling and care during the experiments were conducted according to standard guidelines and the study is reported under ARRIVE guidelines with approval No (202,014) [19].

## Methods

### Plant preparation and standardization

To prepare the Tamarix extract for HPLC analysis, 50 mg of the extract was dissolved in methanol using sonication and the volume was made up to 50 ml. The resulting solution was injected into HPLC in triplicate. For the extraction of the powder, 500 g of it was extracted twice with 2000 ml of methanol. The solvent was then evaporated under reduced pressure, and the extract was stored at 4 °C.

#### Chromatographic conditions

Chromatographic analysis was carried out using Agilent 1260 Infinity Quaternary LC, supplied with Agilent 1260 Infinity; Quaternary Pump (G1311B), and Diode Array Detector (G1315D, VL) coupled to Agilent Open LAB Chem Station B.04.03 software. Analytes were separated on Agilent Zorbax Eclipse XDB-C18 (250 mm× 4.6 mm i.d., 5 μm particle diameter) protected with Agilent Zorbax XDB-C18 pre-column (Agilent Technologies, Palo Alto, CA, USA). The flow rate of 1.0 mL/min and the UV detector was set at 320 nm. Separation was performed with 0.1% trifluoroacetic acid in acetonitrile (**A**) and 0.1% trifluoroacetic acid in water (**B**) and gradient elution program at a flow rate of 1 ml/min starting with 20% **A** in **B** for 5 min, 20–30% A/B in 5 min, 30–50% **A** in **B** in 5 min, 50–100% **A** in **B** in 10 min followed by washing with 100% **A** for 5 min.

#### Establishment of the standard calibration curve

**Fig. 1:**
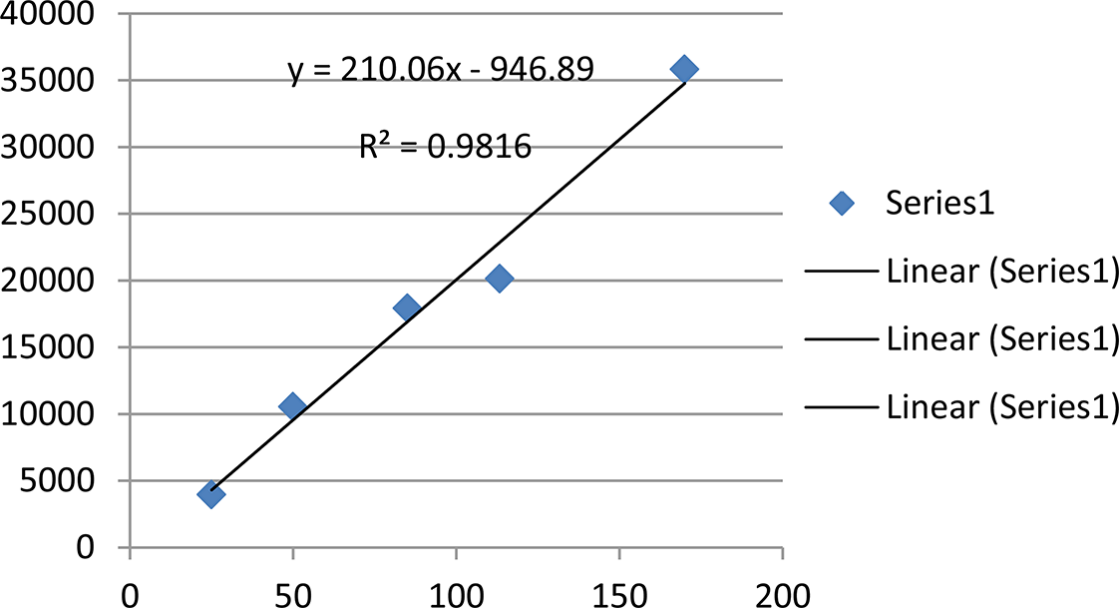
Standard calibration curve of ferulic acid

A standard calibration curve of standard ferulic acid (Fig. 1) was drawn in concentrations ranging from 25.0 to 200 µg/ml.

### Acute oral toxicity

The acute oral toxicity model was carried out according to the method of Organization for Economic Co-operation and Development (OECD) guidelines for the testing of chemicals [20]. After fasting overnight with water ad libitum, five rats were assigned to the experimental and control group. The methanolic extract of TA was administered orally in different graded doses (up to 4,000 mg/kg BW) using rat oral gavage. The animals were monitored for any changes in their autonomic or behavioral responses for four days. An observation was conducted daily to record any death, illness, or treatment-related side effects. The animals were observed for two weeks [21].

### Anti-inflammatory activities

#### In-vivo anti-inflammatory activity

##### Carrageenan-induced paw edema

The potential anti-inflammatory action was measured using a carrageenan-induced rat paw edema assay [22]. The paw inflammation and edema were induced by injecting 0.1 ml of 1% freshly prepared suspension of carrageenan into the right footpad of all rats in the five groups. The left hind paw was left untreated and used as a control to calculate the change in paw thickness. The progression of paw edema was measured at 0, 1, 2, 3, 4, and 5-hour intervals using a digital Vernier caliper. Thirty-animals were divided into five groups, each consisting of six rats. One hour before carrageenan injection, the first three groups were pretreated with TA at doses of 100, 200, and 400 mg/kg respectively. The remaining two groups received distilled water (control) and diclofenac sodium (reference drug).

The percent of the edema’s inhibition was calculated in comparison to the control groups as per the formula shown below:

$$\frac{ \left({\text{Change in control}} - {\text{Change in treatment}}\right)* 100}{{\text{Change in control}}}$$

Where the change of paw thickness values was calculated from the difference between the left and the right paw volumes.

##### Assessment of the IL-1β, TNF-α, NO and COX-2 levels in the rat paw

The levels of TNF-α, IL-1β, NO, and COX2 in the tissue were determined using an enzyme-linked immunosorbent assay (ELISA) method [23]. After the fifth reading, Animals were humanely euthanized by cervical dislocation and the paws were removed. The samples were stored at a temperature of -20 °C to be processed for TNF-α, IL-1β, NO, and COX2 determination. The subcutaneous tissue of the right paw and that surrounding the tarso-tibial joints were homogenized in PBS with a pH of 7.4. The homogenates were then centrifuged at 10,000 rpm for 10 min at a temperature of 4 °C. The levels of IL-1β and TNF-α in the supernatants were determined using a quantitative sandwich enzyme immunoassay technique according to the manufacturer’s instructions. Briefly, the microtiter plate (provided in this kit) has been pre-coated with a specific monoclonal antibody. After pipetting the samples into the wells of this plate, all unbound material was washed away. The target cytokine in standards and samples was caught by the immobilized monoclonal antibody in the plate and a secondary biotinylated polyclonal antibody, which is recognized by a streptavidin-antibody. Subsequently, the developed color was stopped, and the plates were read at a wavelength of 490 nm using an ELISA reader, and standard curves were established to calculate the concentration of each parameter.

#### In-vitro experiments

##### Red blood cells (RBCs) suspension

Blood was obtained from the retro-orbital plexus of healthy rats using the intraperitoneal injection of Ketamine and Xylazine (70 and 10 mg/kg, respectively). Blood was collected in tubes containing heparin to prepare RBC suspension. The tubes were then centrifuged at 3000 rpm for 15 min at room temperature, and the supernatant was removed. The RBCs were washed thoroughly three times using phosphate buffer saline (PBS) with a pH of 7.4. A 10% of the cell suspension was prepared using PBS and used in the assays.

#### Heat-induced hemolysis

The mixture for the reaction (2 ml) contains 1 ml of RBC suspension (10%) and 1 ml of various concentrations of test samples such as standard drugs or plant extract. Control tubes were treated with saline solution. After incubation at 56 °C for 30 min, the tubes were cooled using tap water. Then, the tubes were centrifuged at 2500 rpm for five minutes, and the absorbance of the supernatant was measured at a wavelength of 560 nm [24]. The experiment was performed three times for each of the test samples. The inhibition percentage in tubes was calculated using the following formula:

$$\% \; {\text{inhibition of hemolysis}} = 100-\left[1-\left(\frac{{\text{OD}}2-{\text{OD}}1}{{\text{OD}}3-{\text{OD}}1} \right)\right]$$

Where: OD_1_ = Test sample unheated OD_2_ = Test sample heated and treated OD_3_ = control heated sample.

### Statistical analysis

All data were expressed as mean ± SE and analyzed statistically using Statistical Package for Social Science (SPSS) version 25.0 for Windows (SPSS Inc, Chicago, IL). The statistical significance of differences among different study groups was evaluated by one-way analysis of variance (ANOVA). Differences were considered significant when the p-value was greater than 0.05. To determine differences between means of treatments at significance rates of 0.05, Duncan’s multiple range test was used.

## Results

### HPLC analysis of *Tamarix aphylla* methanolic extract

**Fig. 2:**
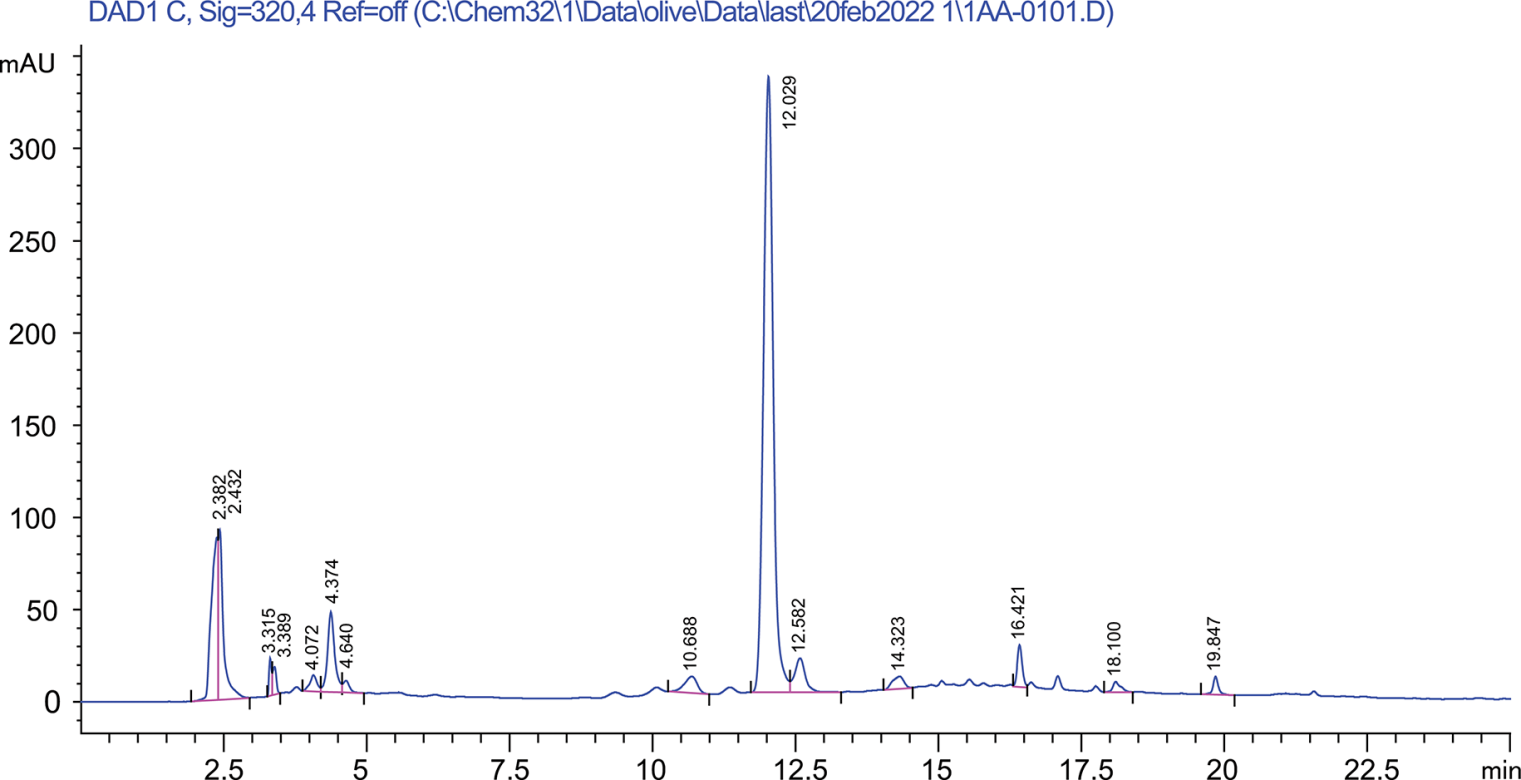
HPLC chromatographic separation of *Tamarix aphylla* methanolic extract

From standard calibration curve and the equation ${\text{Y}}=210.06{\text{X}}-946.89$ the concentration of ferulic acid was determined to be 1.42% (Fig. 2).

### Acute oral toxicity of *Tamarix aphylla* extract

The methanolic extract of *T. aphylla* plants did not cause any toxic symptoms or mortality in Wistar rats when given a single graded dose of up to 4000 mg/kg BW. The animals were observed for 14 days, during which no observable behavioral changes were noted. Additionally, there were no instances of drug-related morbidity or mortality throughout the experiment.

### Anti-inflammatory activities

#### *In-vivo* anti-inflammatory activity

##### Treatment with *Tamarix aphylla* extract significantly reduced the mean paw volume in rats

The thickness of the rat paw was noticeably increased after the intra-planter injection of carrageenan. However, Tamarix aphylla significantly reduced paw inflammation in a dose-dependent manner. Even when compared to the reference drug diclofenac, the extract showed potent effects by reducing paw thickness or inflammation inhibition percentage throughout the five-hour experiment period (Table 1).

**Table 1:** Changes in rats paw thickness in carrageenan-induced inflammation

Group	Dose (mg/kg)	Change in paw thickness in mm (hours post treatment)				
		1st	2nd	3rd	4th	5th
Saline	–	2.28 ± 0.27^a^	2.54 ± 0.39^a^	3.59 ± 0.43^a^	3.22 ± 0.29^a^	3.11 ± 0.68^a^
Diclofenac sodium	10	1.32 ± 0.26^b^ (38.66)	1.29 ± 0.26^bc^ (55.71)	1.31 ± 0.19^cd^ (61.78)	1.36 ± 0.23^c^ (60.51)	1.22 ± 0.28^b^ (63.81)
TA	400	1.09 ± 0.31^b^ (54.62)	0.78 ± 0.24^c^ (67.41)	0.84 ± 0.27^d^ (69.78)	1.11 ± 0.16^c^ (73.63)	1.22 ± 0.23^b^ (70.06)
	200	1.58 ± 0.26^ab^ (35.75)	1.55 ± 0.25^bc^ (43.90)	1.85 ± 0.35^ab^ (50.32)	1.38 ± 0.25^c^ (48.50)	1.23 ± 0.14^b^ (53.47)
	100	1.91 ± 0.20^ab^ (16.56)	1.87 ± 0.14^ab^ (24.88)	2.62 ± 0.13^b^ (24.86)	2.29 ± 0.10^b^ (32.66)	2.07 ± 0.08^ab^ (39.96)

Each value is the mean” S.E.M for 6 mice. Different letters within the count column mean statistical significance at (*P* < 0.05)

The percentage of inhibition values are given in parentheses

##### *Tamarix aphylla* extract significantly reduced the concentrations of tumor necrosis factor-alpha (TNF-α) and interleukin 1-beta (IL-1β) inside the rat paw tissue

**Fig. 3:**
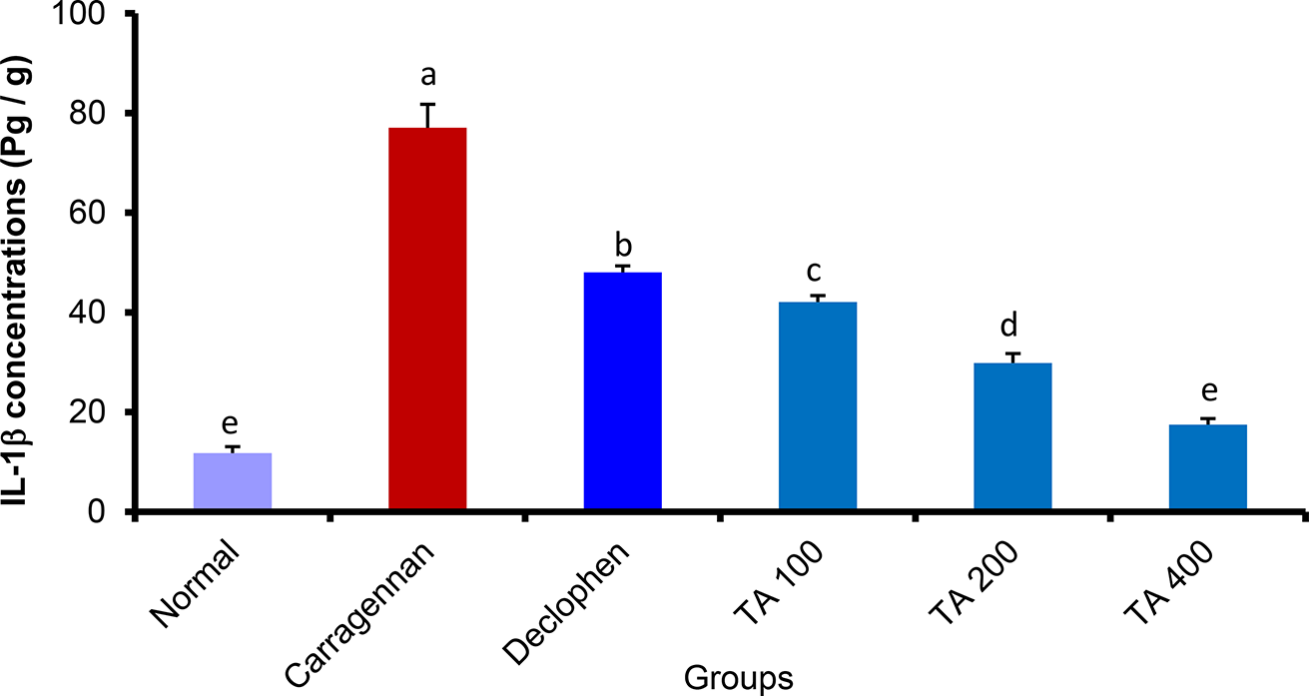
The effect of *Tamarix aphylla* (TA) on IL-1β levels in carrageenan injected paw. Each value is the mean ± S.E.M for 6 rats per group. Above each column, different letters mean statistical significance at (*P* < 0.05)

**Fig. 4:**
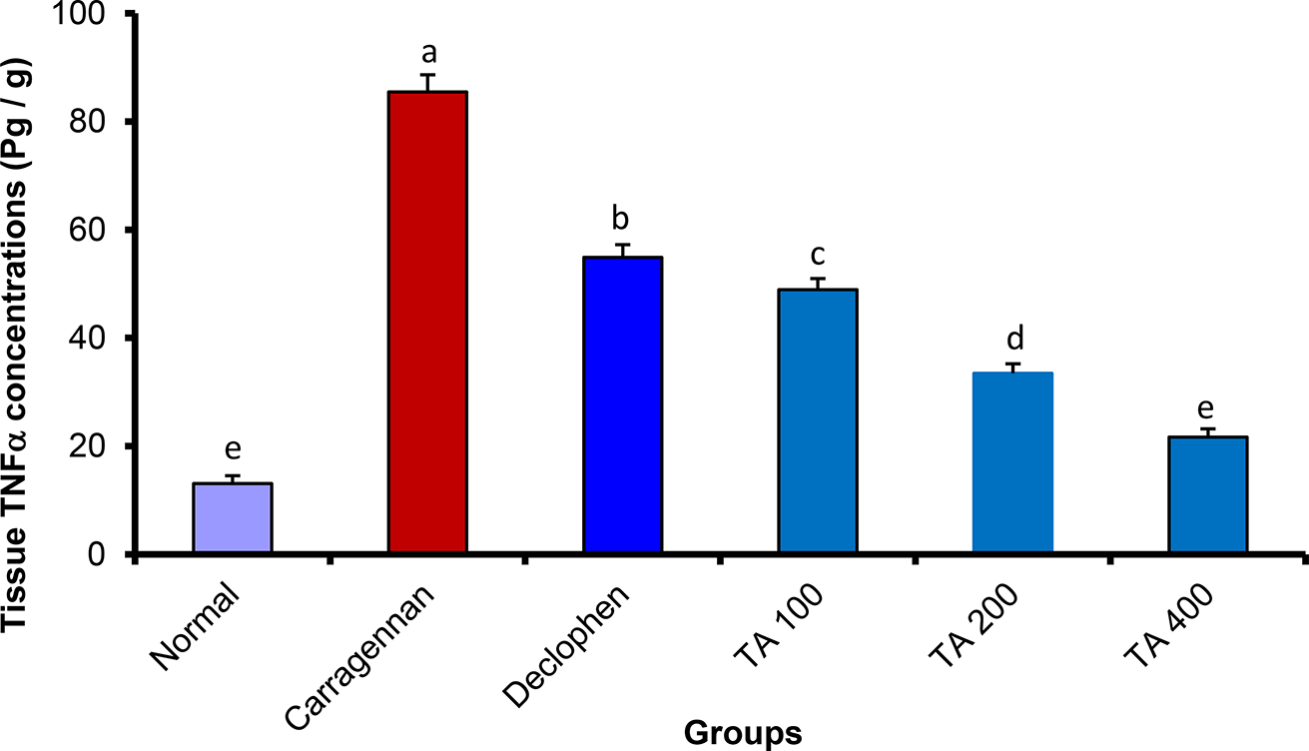
The effect of *Tamarix aphylla* (TA) on TNFα levels in carrageenan injected paw. Each value is the mean ± S.E.M for 6 rats per group. Above each column, different letters mean statistical significance at (*P* < 0.05)

To measure the amount of TNF-α and IL-1β, the rat paw homogenate was utilized. The concentration of IL-1β and TNF-α was substantially increased by the sub-planter injection of carrageenan. However, Tamarix aphylla significantly reduced the concentration of TNF-α (ranging from 21 ± 0.68 to 48.9 ± 2.1) and IL-1β (ranging from 17.45 ± 1.24 to 42.08 ± 1.30) in a dose-dependent manner in tissue (Figs. 3 and 4).

##### Treatment with *Tamarix aphylla* extract substantially reduced cyclooxygenase 2 (COX-2) and nitrous oxide (NO) inside the rat paw tissue

**Fig. 5:**
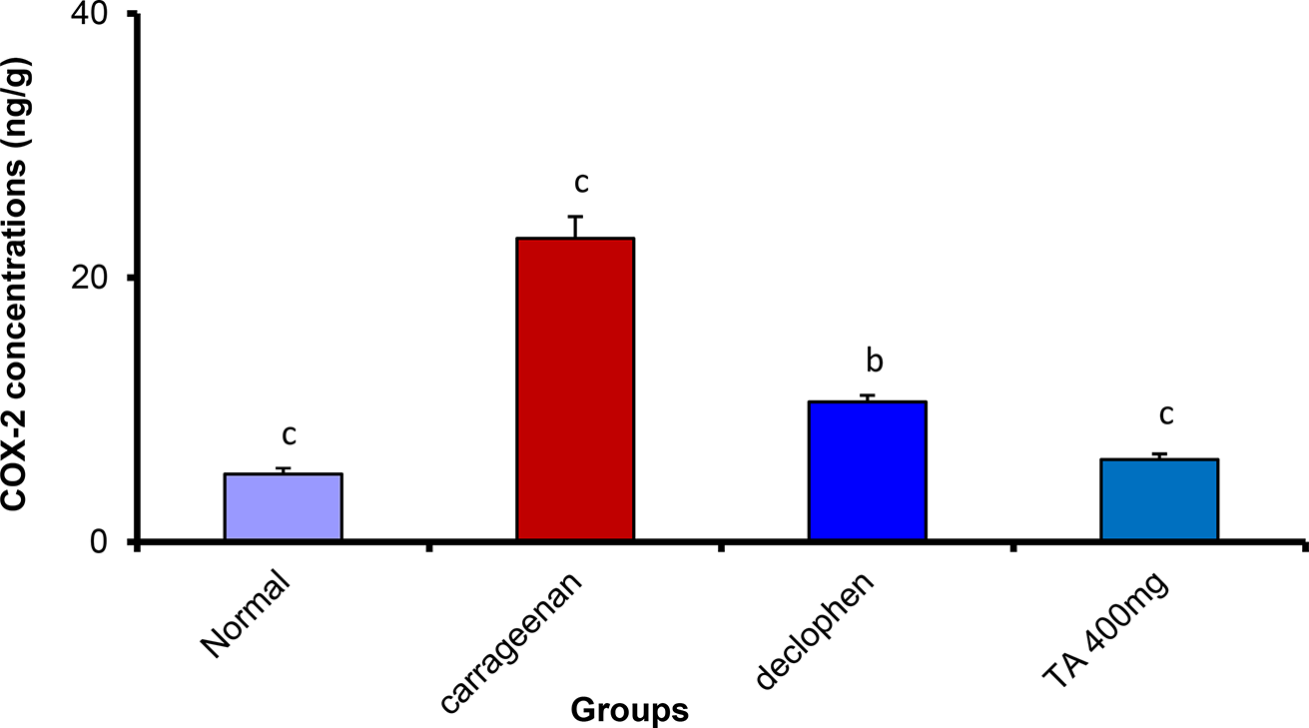
The effect of *Tamarix aphylla* (TA) on COX-2 levels in carrageenan-injected paw. Each value is the mean ± S.E.M for 6 rats per group. Above each column, different letters mean statistical significance at (*P* < 0.05)

**Fig. 6:**
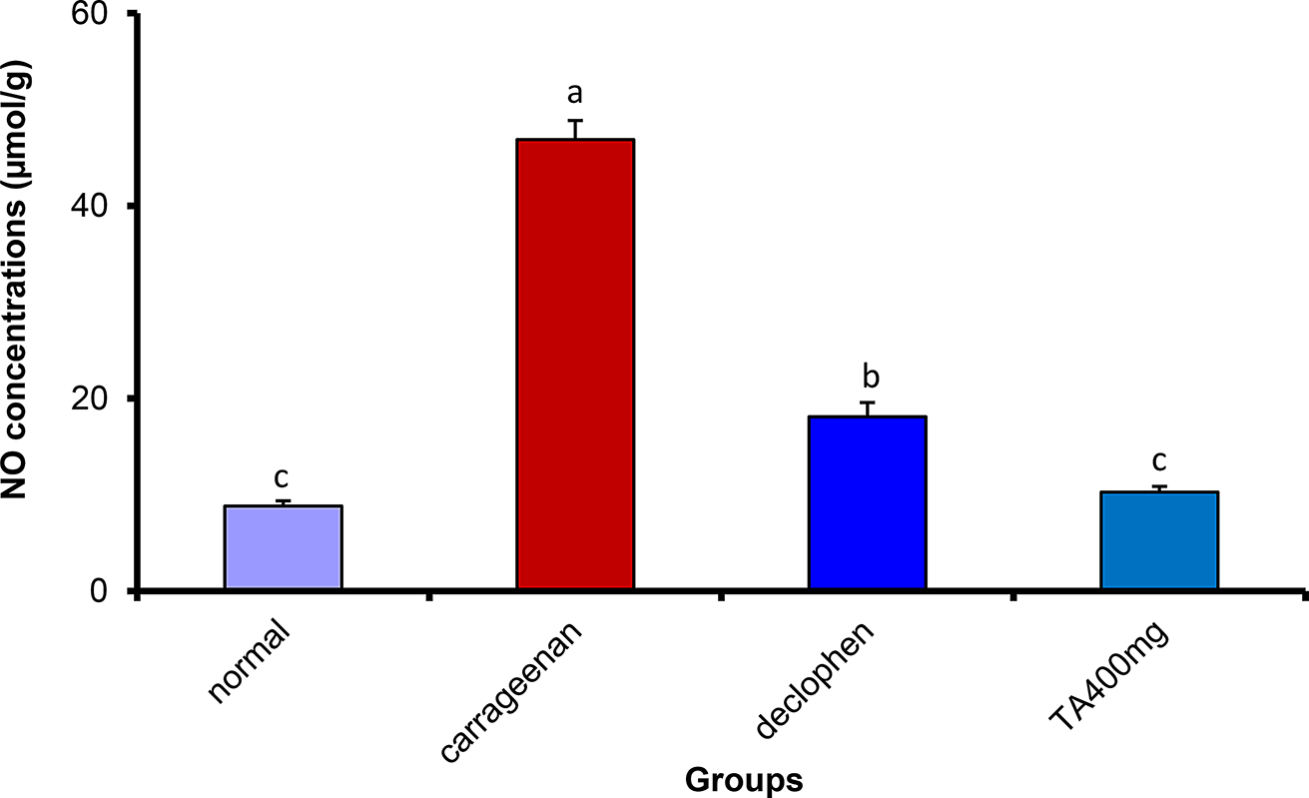
The effect of *Tamarix aphylla* (TA) on NO levels in carrageenan-injected paw. Each value is the mean ± S.E.M for 6 rats per group. Above each column, different letters mean statistical significance at (*P* < 0.05)

The carrageenan treated group showed a significant increase in both COX-2 and NO. However, Tamarix aphylla at a dose of 400 mg/kg resulted in a marked reduction in the level of COX-2 and NO, which was statistically equivalent to the concentration of the normal group. The standard drug (Declophen) also reduced the concentration of both COX-2 and NO to reach 10.60 ± 0.51 and 18.09 ± 1.49, respectively. Nevertheless, the effect of the plant extract was more potent than that of the standard drug (Figs. 5 and 6).

#### *In-vitro* anti-inflammatory

##### *Tamarix aphylla* extract significantly protected the erythrocyte membrane against heat-induced hemolysis

**Fig. 7:**
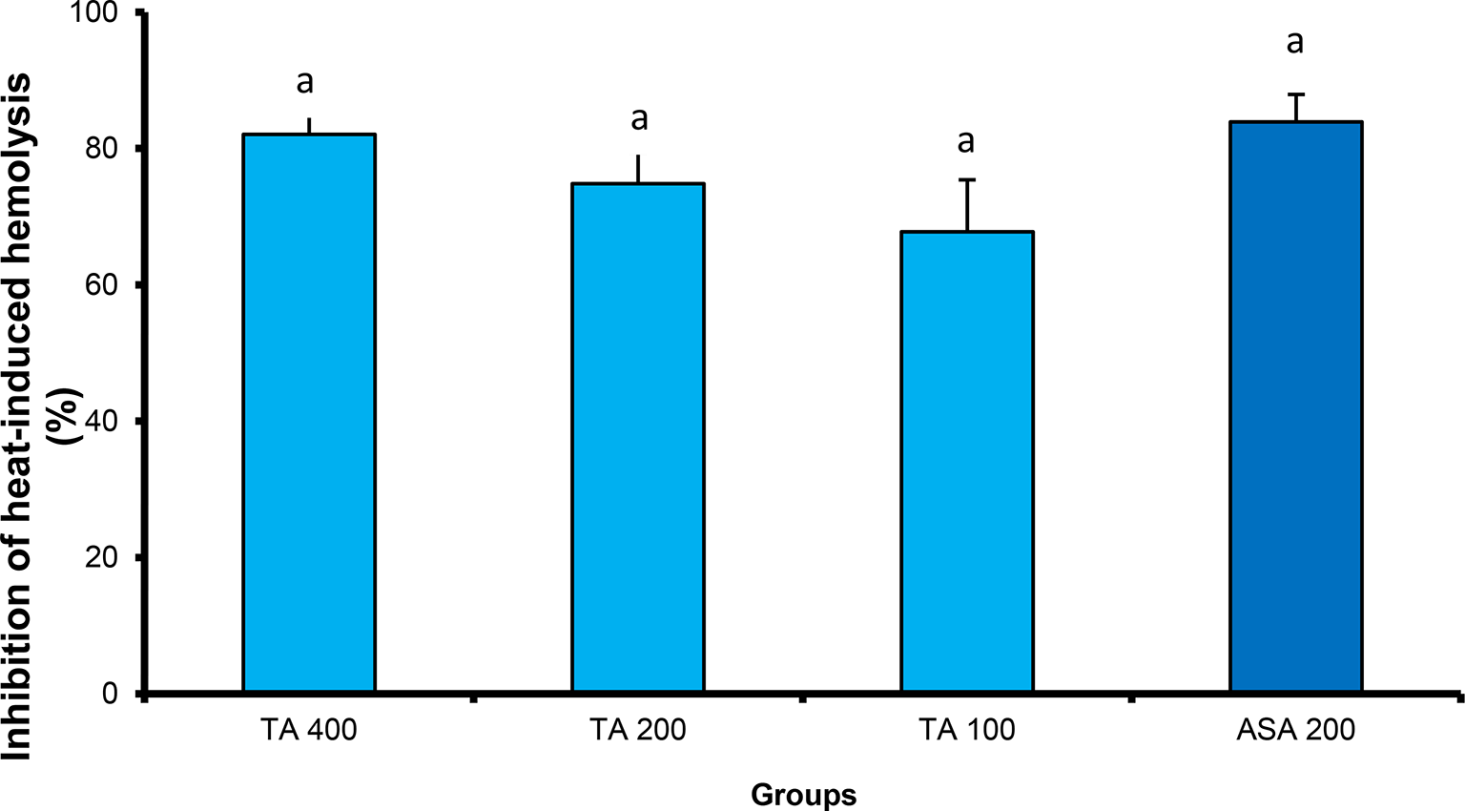
Inhibition percentage of *Tamarix aphylla* (TA) on heat-induced hemolysis. Results are expressed as mean ± SE. Different superscripts, within the same column mean statistical significance at (*P* < 0.05)

The findings of the erythrocyte membrane stabilization test indicated that the plant extract had a potent ability to protect the RBC membrane against heat-induced hemolysis in a dose-dependent manner. When compared to the aspirin-treated group, there were no statistically significant differences between the percentage of inhibition produced by all different doses of Tamarix aphylla and that produced by 200 µg of aspirin. The inhibition percentages recorded for TA extract (100, 200, and 400 µg/kg) were 67.78%, 74.82%, and 82.08%, respectively, compared to 83.89% produced by Aspirin (Fig. 7).

## Discussion

The evaluation of the extract’s acute toxic effect is usually performed as an initial step to avoid the potentially noxious effects of the plant or any of its chemical constituents following the acute oral administration [25]. Besides, Acute oral toxicity studies allow the determination of LD50 of the compound under investigation and permit searching for the biological therapeutic effect of the compound [26, 27]. After performing the acute oral toxicity of the extract, we found that graded doses of up to 4000 mg/kg of the extract did not cause any behavioral changes or lethality within 24 h in Wistar rats. Therefore, it can be concluded that the dose of 4000 mg/kg was well-tolerated and did not produce any toxicological changes in the physical condition and appearance of the rats. These findings are consistent with previous studies, which reported that LD50 of TA is above 1500 mg/kg BW, and revealed that the methanolic extract of TA produced no toxic symptoms in graded doses up to 4000 mg/kg in acute and sub-chronic toxicity studies [28, 29]. Based on OECD guidelines for testing chemicals, the methanolic extract of TA was considered safe [30]. Thus, it is suggested that the oral median lethal dose (LD50) of TA was above 4000 mg/kg. Furthermore, it is suggested that doses of 100, 200, and 400 mg/kg, which are equivalent to 1/40th, 1/20th, and 1/10th of the tested dose of extract (4000 mg/kg), would be safe for further experiments.

Carrageenan-induced inflammation assay was used to evaluate the potential anti-inflammatory effect of the extract. This animal model is widely used for assessing the anti-inflammatory effects of drugs [31, 32]. It is known that acute inflammation caused by carrageenan administration has two phases. The first stage occurs within 1 h and involves histamine, bradykinin, serotonin, and substance P, which cause vascular permeability. The second delayed stage occurs after 1 h and is mainly due to lysosomal enzymes, proteases, and PG release that regulate the excess secretion of inflammatory fluids at the site of inflammation [33]. In this study, paw thickness was measured for five successive hours as an index of increased permeability and edema. The extract from T. aphylla showed anti-inflammatory effects starting from the first measurement of paw thickness, indicating its ability to fight against both the earliest and delayed stages of inflammation. The anti-inflammatory activity of TA was potent and tended to exceed that of the reference drug, especially at the dose of 400 mg, which exceeded the anti-inflammatory effect of diclofenac sodium by around 120%. This effect continued throughout the measurement period. This response tendency of the extracts in carrageenan-induced paw edema indicates good peripheral anti-inflammatory properties of the methanolic extract of TA. This anti-inflammatory effect may be due to the presence of flavonoids. It has been reported that several flavonoids possess anti-inflammatory [34]. Flavonoids are known to inhibit the enzyme prostaglandin synthetase, more specifically the endo-peroxidase and are reported to produce an anti-inflammatory effect. Further laboratory analysis was conducted to investigate the potential anti-inflammatory mechanism through measuring the levels of IL-1β, TNF-α, COX-2 and NO.

In this study, ELISA results showed that the plant extract significantly reduced all pro-inflammatory markers that were measured. Interestingly, the reduction was slightly higher than that exerted by the standard drug (diclofenac), which validates the anti-inflammatory potential of the extract and suggests a possible mechanism by interfering with some inflammatory mediators and attenuating inflammation progression. These results are consistent with other studies [15, 35].

It is previously documented that the protection of a lysosomal membrane can play an important role in preventing the release of lysosomal enzymes and reducing inflammatory events. This study revealed that the methanolic extract of TA has the ability to stabilize the rat’s RBC membrane. T. aphylla can protect the lysosomal membrane against damage, which in turn prevents the release of lysosomal enzymes and inflammatory mediators, thus contributing strongly towards its anti-inflammatory action [36].

## Conclusion

We can attribute the potent anti-inflammatory activity of *T. aphylla* to its ability to protect the lysosomal membrane and decrease the levels of pro-inflammatory mediators at the site of inflammation. The results of this study suggest that *T. aphylla* could be a good candidate for the management of inflammation.

### Electronic supplementary material

Below is the link to the electronic supplementary material.

**Supplementary Material 1**: A schematic diagram for the experimental design used in the current study

## Data Availability

We confirm that all the data supporting the finding are available in the article and its supplementary data.
