# Supplementary material for: Involvement of TNFα, IL-1β, COX-2 and NO in the anti-inflammatory activity of Tamarix aphylla in Wistar albino rats: an in-vivo and in-vitro study
Source: BMC Complement Med Ther. 2024 Jan 25;24:57. doi: 10.1186/s12906-024-04359-8 (PMC10809683; doi:10.1186/s12906-024-04359-8)
Supplement: Supplementary file 1 — Supplementary Material 1: A schematic diagram for the experimental design used in the current study [file 12906_2024_4359_MOESM1_ESM.docx]

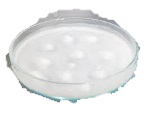


**paw thickness**


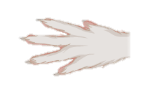


**0.1 ml of 1% carrageenan**


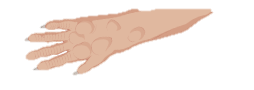

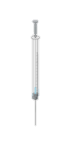

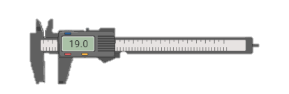


**One hour later**


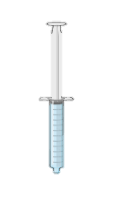

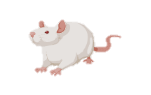


**At doses of 100, 200 & 400 mg/kg**

**TA**


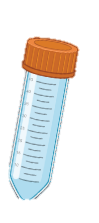


**Declophen**

**(10 mg/kg)**

**One hour later**


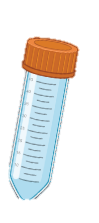


**Five hours**


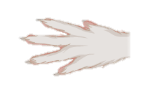


**Inflamed paw**


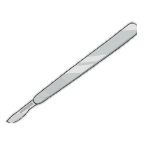

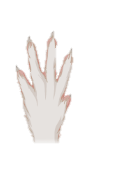

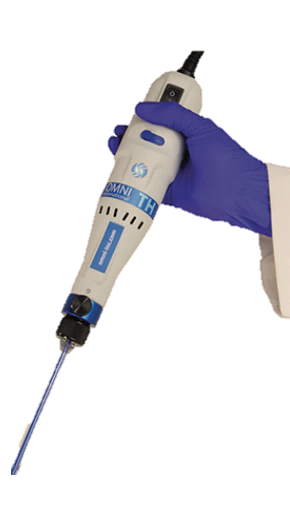

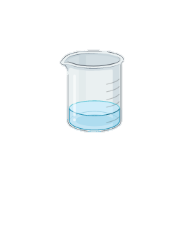

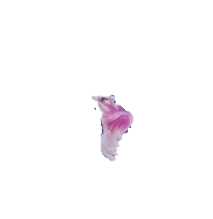

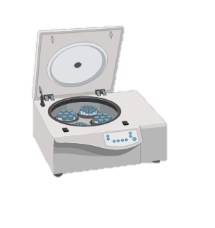

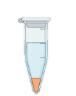

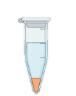

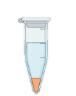

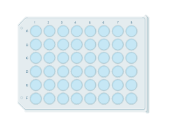

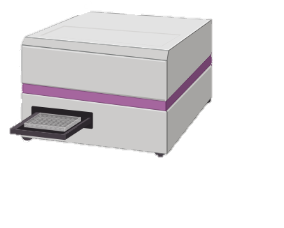

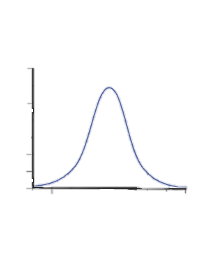


**TNF-α**

**IL-1β**

**COX-2**

**NO**


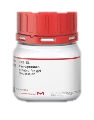

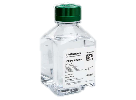


PBS

**Supplementary figure (1): A schematic diagram for the experimental design used in the current study.** TA: *Tamarix Aphylla*, PBS: Phosphate buffer saline, TNFα: Tumor necrosis factor alpha, IL-1β: inter leukin one beta, Cox-2: cyclo-oxygenase two, NO: nitric oxide
